# Effects of Whole-Body Vibration Therapy in Weight-Bearing and Non-Weight Bearing Positions for Upper and Lower Extremities on Balance and Cervical Joint Position Sense in Children With Cerebral Palsy

**DOI:** 10.7759/cureus.62481

**Published:** 2024-06-16

**Authors:** Syed Ali Hussain, Mohammad Reza Hadian Rasanani, Zainab Hassan, Azadeh Shadmehr, Saeed Talebian, Mubin Mustafa Kiyani

**Affiliations:** 1 School of Rehabilitation, Tehran University of Medical Sciences, Tehran, IRN; 2 Shifa College of Medical Technology, Shifa Tameer-e-Millat University, Islamabad, PAK

**Keywords:** proprioception, whole-body vibration therapy, physical therapy, lower extremities, upper extremities, weight-bearing, vibration therapy, joint position sense, cerebral palsy, balance

## Abstract

Introduction: Cerebral palsy (CP) is a complex pathological entity that affects muscular control, coordination, proprioception, fine and gross motor abilities, position, stability, and, in some cases, cognition. This study aimed to compare the effects of whole-body vibration therapy (WBVT) in weight bearing and non-weight bearing positions for the upper and lower extremities on balance and cervical joint position sense in children with spastic CP.

Methods: A randomized controlled trial was carried out on 60 hemiplegic children with spastic CP aged 5-15 years. Following randomization, all participants were allocated into six equal-sized groups based on the application of WBVT for upper extremities, lower extremities, or both simultaneously in either weight-bearing or non-weight-bearing positions. Pediatric balance scale (PBS) and laser tracker system were used to assess functional balance and cervical joint position sense.

Results: One-way analysis of variance for Inter-group analysis showed a statistically significant difference among all groups in PBS and cervical joint position sense (p<0.05).

Conclusion: WBVT was found to be beneficial in improving balance and cervical joint position sense in both weight-bearing and non-weight-bearing positions for the upper and lower extremities in children with cerebral palsy. However, the simultaneous application of WBVT in weight-bearing positions for both upper and lower extremities showed the most significant improvements in improving both balance and cervical joint position sense, indicating the most efficacious position of this treatment approach in children with cerebral palsy.

## Introduction

Cerebral palsy (CP) is a broad term encompassing a group of disorders of movement, balance, posture, and function. It is a non-progressive condition that occurs during fetal brain development or after birth [1]. There are various ways to classify CP including muscle tone, extremity involvement, and site of lesion within the central nervous system [2]. CP has a prevalence of two cases per 1,000 live-born neonates. It has been reported that the spastic diplegic CP is the commonest of all [3].

Children with diplegic CP present with visible deformities including but not limited to hip (anterior titling, internally rotated, adducted), flexed knees, and plantarflexed feet. The shoulder joint is rotated internally, elbows, wrists, hands, and fingers flexed with adducted thumbs. They also show poor balance and movement strategies [4]. Weak and imbalanced muscles are an important factor causing ambulation difficulties. This leads to atrophy and ultimately ortho-myogenic contractures [5]. Due to poor postural control, the children show limited reactive balance. These are the multifactorial issue like abnormal muscle tone, ortho-myogenic contractures, limited degree of freedom, and increased reaction time [6].

Children with CP cannot utilize their proprioceptive senses to control their movements due to confusion caused by excessive amounts of postural tension. The elevated tension creates functional problems in the spindles of the muscle, resulting in insufficient input about movements. That is, instability of trunk muscles weakens the neck flexors and impedes the learning of localization functions, leading to a deficit of head control and asymmetrical postures that create major issues in their capacity to maintain balance [7].

Using new research methods of physical therapy rehabilitation is a must for spasticity management, and improvement of flexibility, movement ability, and fine and gross motor skills. As mentioned, a comprehensive physical rehabilitation program including functional training, muscle lengthening, and relaxation exercises has been considered to be a vital part of managing hypertonicity [8].

Motor functions and bone development can be achieved by muscle-strengthening exercises in CP. One of the safest approach to achieve these involves the application of whole-body vibration (WBV) [9]. This therapeutic modality is proposed to improve muscle strength, balance, gross motor function, and functional performance [10]. Low amplitude with high-frequency vibration therapy improves physical fitness [11]. The subject stands on the vertical oscillating platform to receive WBV therapy (WBVT) and the frequency used ranges 10-25 Hertz. This stimulates the primary and secondary endings of the muscle spindles and activates the motor neuron to induce muscle contraction, similar to the tonic vibration reflex (TVR).The low-frequency training (20 Hz) reduces while high-frequency training (40 Hz) increases muscle tonicity [12]. Therefore, WBV induces positive effects on muscle performance. The muscle spindles are stimulated with WBV resulting in reflex muscle contraction. The muscle spindle induces alpha motor neuron activity according to the TVR. It has been advocated that if active muscle contractions are coupled with these reflexive contractions, it improves motor function. [13] According to a review study by Duquette et al., WBVT improves strength, reduces spasticity, and enhances coordination in children with CP [9].

A study by Trans et al. used two types of platforms; a stable platform (VibM) for WBV exercise which produced increased muscle strength, and the balance board (VibF) produced a better proprioception threshold to detect the passive movement (TDPM) [14]. Both types of therapies produced positive results. A meta-analysis of the effects of WBVT on functional measurements in children with CP showed that this therapy can be used as an alternative method in addition to routine physical therapy for the management of children with CP. It reported improved gait speed and standing function [10]. WBVT is safe, feasible, and acceptable for all age groups including children, adolescents, adults, and geriatric population [15]. Another study reported that a single session of WBVT can improve gait speed, step and stride length, and dynamic ankle range of motion in CP adults. This makes the CP adults more independent and in control of improving or maintaining their ambulation skills. It is a fairly inexpensive intervention that can be used by the physical therapists [16].

In previous studies reporting the immediate and long-lasting effects of WBVT on gait, walking speed, standing function, spasticity, flexibility, strength, proprioception, and range of motion, there have only been measurements related to the lower extremity's function in a weight-bearing position. Hence, a standardized and quantitative measurement for the effect of WBVT applied to the lower extremity and its effect on upper extremities and vice-versa on balance and joint position sense remains unclear in patients with CP. It is reported in the scientific literature that there is an effect of training of lower extremities on upper extremities as well, which is known as the cross-training effect. To the best of our knowledge, there is no study available that examined this effect in the CP population. Therefore, this study aimed to compare the effects of WBVT in weight-bearing and non-weight-bearing positions for the upper and lower extremities on balance and cervical joint position sense in children with CP. The findings of this study will highlight the necessity of considering holistic methods of therapy, targeting multiple limb domains to maximize functional outcomes and promote the well-being of CP children.

## Materials and methods

This double-blinded randomized controlled trial (RCT) was carried out at the Tehran University of Medical Sciences (TUMS), Tehran, Iran. Ethical approval was given by the School of Nursing and Midwifery & Rehabilitation, TUMS Research Ethics Committees (approval number: IR.TUMS.FNM.REC.1402.024) and the clinical trial was registered at the Iranian Registry of Clinical Trial (registration number: IRCT20090301001722N27). The study included 60 children with spastic hemiplegic CP; 30 of them were males and 30 were females.

All participants were screened to assess eligibility, which involved being of both genders of age between five and 15 years, diagnosed with spastic hemiplegic CP and having spasticity scores of 1, 1+, and 2 on the modified Ashworth scale, as well as gross motor function measure levels 1 and 2. Children having no fixed musculoskeletal deformities, history of recent surgery (<1 year ago), unhealed fractures, seizures, auditory or visual issues, botulinum toxin therapy were included in the study. Children with other kinds of CP (ataxic, flaccid, and dyskinetic) or sensory loss in the upper and lower extremities, whose caregivers refused to provide consent, or who were not interested in finishing the course of treatments were excluded from the study. The participation in the research was entirely voluntary. Written consent was obtained from the parents or guardians of all the participants. 

Data was collected from the Pakistan Airforce Base School for Persons with Special Needs, Nur Khan, Rawalpindi, Pakistan. A dedicated room was utilized for this purpose. All the participants, their teachers, and parents or guardians received standard instructions about the procedure. An interview and examination were carried out to seek informed consent after matching the inclusion criteria. After obtaining informed consent from the parents/guardians of the children, a senior physical therapist did the screening examination. Based on the selection criteria, 60 children with spastic hemiplegic CP were recruited in the study. After enrolment, all participants were issued serial numbers (ranging from 1 to 60) and separated into six groups (Aa, Ab, Ac, Ba, Bb, and Bc), with a male-to-female ratio of 1:1. Randomization was carried out using Random Allocation Software (Version 1.0) created by the Department of Anesthesia at Isfahan University of Medical Sciences in Isfahan, Iran. A timetable was developed for the interventional phase of the study, and parents were asked to make sure that children were present during that time to avoid any missed sessions.

In this double-blinded study, caregivers were blinded to the study groups, and outcome assessors were blinded to the research hypothesis and intervention protocols. Two physical therapists, already working in the setting, participated in providing care and assessing outcomes. Each physical therapist assessed participants of the opposite gender in the presence of a school-maid. They then provided the treatment protocol, shared by the study's primary investigators, to participants of the same gender.

Procedure

All participants received treatments three days a week for four consecutive weeks, totaling 12 sessions. The treatment frequency for all groups was 18 Hertz, with an amplitude of 12 mm. The vibration frequency was increased by 1 Hertz per two seconds until reaching 18 Hertz. Each session consisted of three minutes of vibration therapy followed by two minutes of rest, repeated four times during the 20-minute treatment. All groups received WBV training for 20 minutes a day, three days a week for four weeks. The groups received vibration therapy in different positions.

Group Aa (WBVT in Weight-Bearing for Upper Extremities Only)

The patients assumed a standing position and placed their both hands on the whole-body vibration platform, such that their elbow joints were slightly flexed.

Group Ab (WBVT in Weight-Bearing for Lower Extremities Only)

Patients were given instructions to stand on the vibration platform. They stood barefoot, with feet parallel and knees slightly flexed (30°). When standing, the feet were evenly spaced from the device's center line.

Group Ac (WBVT in Weight-Bearing for Both Upper and Lower Extremities Simultaneously)

The patient assumed a standing position on the WBV platform, and placed both hands on the second WBV platform, such that their elbow joints were slightly flexed.

Group Ba (WBVT in Non-Weight-Bearing for Upper Extremities Only)

The patients were seated on the chair and the WBV platform was placed on a couch so that they could place both their hands comfortably on to the platform such that there was no weight-bearing. The elbows were flexed to 90°.

Group Bb (WBVT in Non-Weight-Bearing for Lower Extremities Only)

The patients were seated on the chair and placed their both feet on the WBV platform comfortably such that there was no weight-bearing. The hip and knee joints were placed in 90° of flexion.

Group Bc (WBVT in Non-Weight-Bearing for Both Upper and Lower Extremities Simultaneously)

The patients were in a seated position and placed their both feet on one whole-body vibration platform, and they placed their both hands resting comfortably on the second whole-body vibration platform such that there is no weight bearing. The elbows, hip, and knee were kept at 90° of flexion.

Additionally, all participants received conventional physical therapy, including stretching exercises, parallel bar exercises, and wobble board exercises for 15 minutes per day, as they had received before the study. 

**Figure 1:**
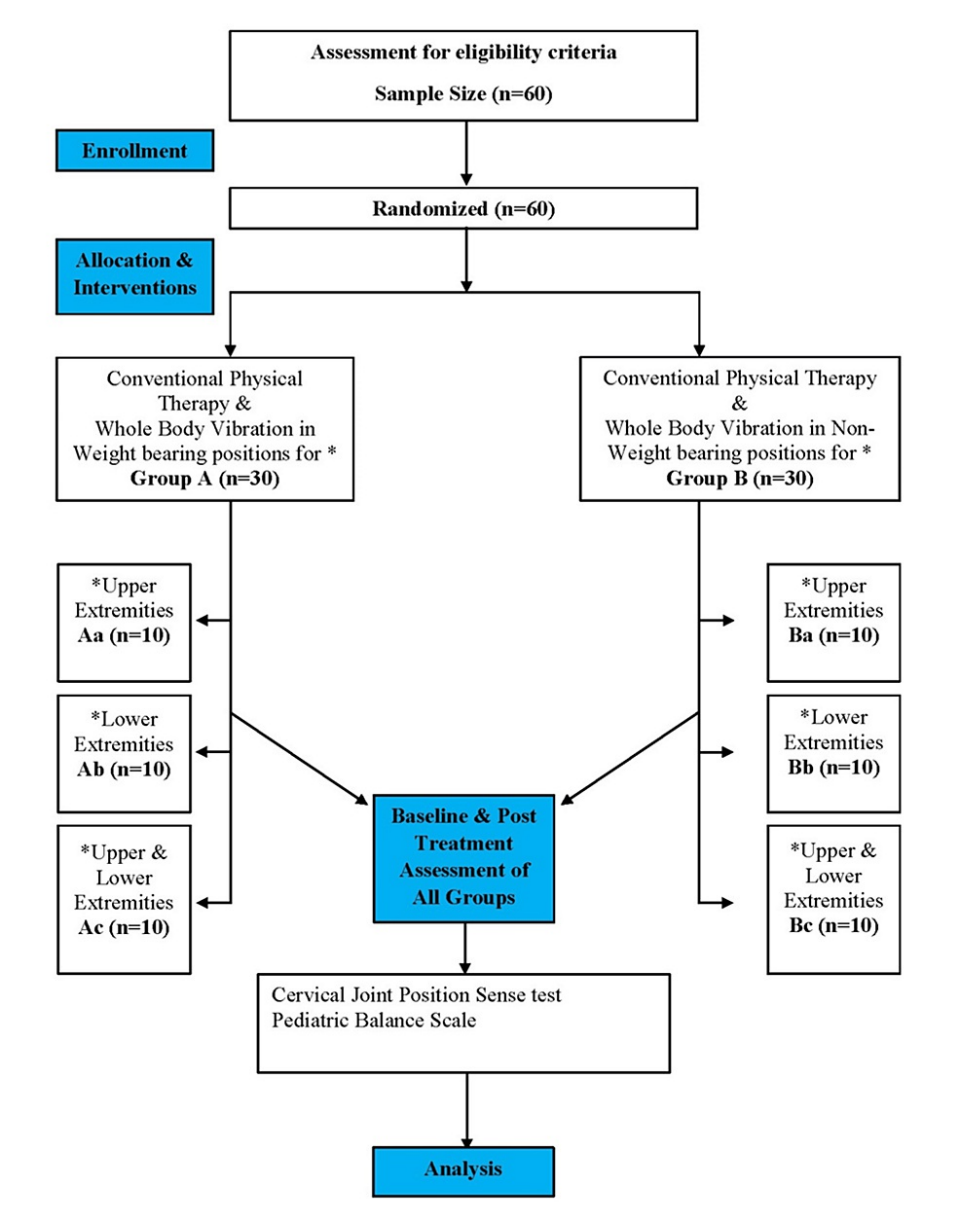
CONSORT Flow Chart CONSORT: Consolidated Standards of Reporting Trials

Figure 1 shows the Consolidated Standards of Reporting Trials (CONSORT) flow chart of the study.

The Pediatric Balance Scale (PBS) was used to assess functional balance. All the procedures were explained and demonstrated until they understood it and one practice session was done before the actual readings were taken. It is a 14-item scale and the score ranges from 0 to 56. For each item, the score ranges from 0 (lowest function) to 4 (highest function). The PBS evaluates a wide range of functional skills necessary for a child to function effectively and safely in the home, classroom, and community. These skills include stepping, reaching forward, reaching to the floor, turning, and stepping on and off of a raised platform. Administering it takes around 20 minutes. Test-retest and inter-rater reliability of this instrument is demonstrated when it is administered to school-age children with mild to moderate motor impairment. The test and retest reliability of the PBS shows an intra-class correlation coefficient of 99% [17].

Cervical joint position sense was assessed by using the laser tracker system. It is used to examine the individual’s head relocation ability; it is a very good indicator of proprioception. We used a laser tracker and a target for assessment. The process was explained and demonstrated to all the participants until they understood it completely and one practice session was done before the actual readings were taken. The distance from the laser to the target chart was 90 cm. The measurements were taken in a seated position. A total of three trials were recorded for the movements of flexion, extension, right and left side bending, and rotations. The best scores were selected for analysis. The test and retest reliability of the PBS shows an intraclass correlation coefficient of 68% [18].

Statistical analysis

IBM SPSS Statistics for Windows, Version 27.0 (Released 2020; IBM Corp., Armonk, New York, United States) was used for the statistical analysis. The normality of data distribution was determined by performing the Shapiro-Wilk test. One-way ANOVA was used for the inter-group comparison of the six groups. The level of significance was considered as 0.05. 

## Results

This study recruited 60 children with cerebral palsy, which were divided into six groups, 10 in each group. The mean age of the participants in Groups Aa, Ab, and Ac was 11.80 ± 1.75 years, 11.90 ± 1.52, and 12 ± 1.88 years, respectively, and the mean age of the participants in Groups Ba, Bb, and Bc was 11.60 ± 1.83 years, 12.10 ± 1.66 years, and 12.30 ± 1.88 years, respectively. Each group comprised an equal number of male and female children (five males and five females). A one-way ANOVA revealed no significant disparity in age across all groups with a p-value greater than 0.05 (Table 1).

**Table 1:** Age distribution UE: upper extremity; LE: lower extremity; WBVT: whole-body vibration therapy

Groups		N	Mean ± SD	p-value
Aa	WBVT for UE only in weight-bearing position	10	11.80±1.75	0.965
Ab	WBVT for LE only in weight-bearing position	10	11.90±1.52	
Ac	WBVT for both UE and LE in weight-bearing position	10	12.00±1.88	
Ba	WBVT for UE only in non-weight-bearing position	10	11.60±1.83	
Bb	WBVT for LE only in non-weight-bearing position	10	12.10±1.66	
Bc	WBVT for both UE and LE in non-weight-bearing position	10	12.30±1.88	

Of the children in Group Aa, 90% had the right side of their body affected and 10% had the left side affected. In Group Ab, 30% had their left side affected, 70% had their right side affected, and in Group Ac, both sides were equally affected. In Groups Ba and Bb, 60% had the right side affected and 40% had the left side affected and in Group Bc, 80% had the right side affected and 20% had the left side affected body (Table 2).

**Table 2:** Frequency and percentage of the affected side UE: Upper extremity; LE: Lower extremity; WBVT: whole-body vibration therapy

Groups		N	Affected Side, n (%)	
			Right	Left
Aa	WBVT for UE only in weight-bearing position	10	9 (90)	1 (10)
Ab	WBVT for LE only in weight-bearing position	10	7 (70)	3 (30)
Ac	WBVT for both UE and LE in weight-bearing position	10	5 (50)	5 (50)
Ba	WBVT for UE only in Non-weight-bearing position	10	6 (60)	4 (40)
Bb	WBVT for LE only in non-weight-bearing position	10	6 (60)	4 (40)
Bc	WBVT for both UE and LE in non-weight-bearing position	10	8 (80)	2 (20)

Table 3 shows the inter-group comparison of the PBS and test statistics of one-way ANOVA. The pre-treatment p-value for the PBS was 0.69, indicating that there was no significant disparity between the groups at baseline. The post-treatment p-value for the PBS was 0.000, indicating that there was a significant disparity across the groups after treatment. The post hoc analysis revealed that all the ranges in Group Ac improved more significantly than other groups.

**Table 3:** Inter-groups comparison of Pediatric Balance Scale (One way ANOVA) *shows significant difference between groups on the post hoc test

Balance		Aa	Ab	Ac	Ba	Bb	Bc	p-value
Pre	Mean ± S.D.	25.50±2.50	26.70±3.02	26.20±2.57	25.10±2.42	25.50±2.63	25.00±2.86	0.69
Post	Mean ± S.D.	28.70±3.52	34.50±3.06	39.60±3.23*	27.80±1.93	31.30±2.75	35.20±3.88	0.00

Table 4 shows the inter-group comparison of cervical joint position sense and test statistics of ANOVA. The pre-treatment p-value for cervical flexion was 0.84, indicating that there was no significant disparity across the groups before the treatment. The post-treatment p-value for cervical flexion was 0.000, indicating that there was a significant disparity across the groups after the four weeks of treatment. The pre-treatment p-value for cervical extension was 0.95, indicating that there was no significant disparity across the groups before the treatment. The post-treatment p-value for cervical extension was 0.000, indicating that there was a significant disparity across the groups after the four weeks of treatment.

**Table 4:** Inter-group comparison of cervical joint position sense (one way ANOVA) * shows significant difference between groups on the post hoc test

Joint Position Sense in Degrees				Aa	Ab	Ac	Ba	Bb	Bc	p-value
Cervical	Flexion	Pre	Mean ± S.D.	11.71±0.74	11.81±0.47	11.89±0.18	11.64±0.93	11.62±1.22	11.42±0.86	0.84
		Post	Mean ± S.D.	9.82±0.86	9.98±0.53	8.54±0.66*	10.88±0.78	10.57±1.43	10.04±0.81	0.00
	Extension	Pre	Mean ±S.D.	11.76±1.32	11.42±1.20	11.51±1.29	11.34±0.09	11.62±1.05	11.37±1.11	0.95
		Post	Mean ± S.D.	10.10±1.32	10.60±1.11	8.40±1.07*	10.00±0.43	10.85±0.97	9.83±0.99	0.00
	Right Rotation	Pre	Mean ± S.D.	12.30±1.02	12.43±1.22	12.15±1.54	12.39±1.03	12.17±1.05	12.34±1.49	0.99
		Post	Mean ± S.D.	10.00±1.03	11.25±1.13	8.82±1.60*	10.86±0.79	11.03±0.91	10.61±1.25	0.00
	Left Rotation	Pre	Mean ± S.D.	11.99±1.28	12.09±1.52	12.35±1.39	12.24±0.77	12.06±0.95	12.12±1.18	0.98
		Post	Mean ± S.D.	10.24±0.96	10.89±1.19	9.14±0.86*	10.67±0.54	11.04±1.02	10.47±1.11	0.001
	Right Side Bending	Pre	Mean ± S.D.	11.99±1.28	12.09±1.52	12.35±12.35	12.24±0.77	12.06±0.95	12.12±1.18	0.98
		Post	Mean ±S.D.	10.24±0.96	10.89±1.19	9.14±0.86*	10.67±0.54	11.04±1.02	10.47±1.11	0.001
	Left Side Bending	Pre	Mean ±S.D.	12.43±0.99	12.25±0.84	12.05±0.95	12.26±1.02	12.19±0.97	12.38±0.95	0.96
		Post	Mean ± S.D.	10.35±0.99	10.94±0.82	9.08±0.90*	10.82±0.79	11.28±0.73	10.52±0.95	0.00

The pre-treatment p-value for cervical right rotation was 0.99, indicating that there was no significant disparity across the groups before the treatment. The post-treatment p-value for cervical right rotation was 0.000, indicating that there was a significant disparity across the groups after the four weeks of treatment. The pre-treatment p-value for cervical left rotation was 0.98, indicating that there was no significant disparity across the groups before the treatment. The post-treatment p-value for cervical left rotation was 0.001, indicating that there was a significant disparity across the groups after the four weeks of treatment.

The pre-treatment p-value for cervical right-side bending was 0.98, indicating that there was no significant disparity across the groups before the treatment. The post-treatment p-value for cervical right-side bending was 0.001, indicating that there was a significant disparity across the groups after the four weeks of treatment. The pre-treatment p-value for cervical left-side bending was 0.96, indicating that there was no significant disparity across the groups before the treatment. The post-treatment p-value for cervical left-side bending was 0.00, indicating that there was a significant disparity across the groups after the four weeks of treatment.

The post hoc analysis revealed that all the ranges in Group Ac improved more significantly than other groups.

## Discussion

The results of the present study showed that WBVT had beneficial effects in improving balance and cervical joint position sense in both weight-bearing and non-weight-bearing position for the upper and lower extremities in children with CP. Notably, the simultaneous application of WBVT in weight-bearing position for both upper and lower extremities showed the most significant improvements in improving both balance and cervical joint position sense, indicating the most efficacious position of this treatment approach in children with CP.

Ko et al. explored the impact of WBVT combined with traditional physiotherapy on joint position sensing and balance in 24 cerebral palsy children [19]. The frequency of the vibrations in their investigation was adjusted to 20-24 Hz, with an amplitude of 1-2 mm. The study found that three weeks of WBVT improved ankle joint position sensing and gait characteristics in children with CP. Similarly, in our study, the balance and joint position sensing of cervical flexion, extension, left and right rotation, and left and right lateral flexion improved significantly after the treatment of WBVT for four weeks in both weight-bearing and non-weight-bearing positions for the upper and lower extremities in children of CP. Another study demonstrated that including WBVT with head and neck retraction exercise improved cervical joint position sense [20].

Proprioception deficiencies in CP are likely caused by central nervous system deficits that disrupt all proprioceptive signals to the cortex, including muscle spindles, Golgi tendon organs, and sensory afferent stimulation of skin and joints. Spastic fibers of muscle and shorter sarcomeres among individuals with CP may negatively impact joint position sensing by reducing and tightening the muscle tissue, influencing the interaction between joints and muscles, and causing disruption in the sensitivity of muscle spindles. Joint position sensing helps manage posture by detecting joint movement [21]. The fundamental mechanism by which WBVT can affect neuromuscular responses has been explained in several different contexts. Vibration within the muscles causes alterations to the tension of the intrafusal fibers, which in turn causes Ia-afferent excitation and reflex muscular contraction. The co-activation of antagonist and agonist as well as the acquisition of motor units can both be enhanced by the tonic vibration reflex via activating muscle spindles and poly-synaptic routes. Enhanced stiffness of the muscles and action, which are known to improve joint proprioception, maybe the collective outcome of these reactions [13].

A study by Jones et al. investigated the impact of only one session of WBVT on balance and joint position sense in 36 community-dwelling older adults over the age of 50 [22]. The study found that a single 10-minute session of WBV at 6 Hz and 5 mm peak-to-peak amplitude improved single-limb balance but exhibited no influence on joint position perception. In our investigation, the treatment frequency of WBVT for all groups was 18 Hz with an amplitude of 12 mm. In contrast to their findings on joint position sense, a significant improvement was seen in our study on cervical joint position sense in all groups. This contradiction might be because the use of a low frequency of 6 Hz in their study for only a single session might not be enough to produce a pronounced effect in the joint position sense.

A pilot study by Han et al. was performed to assess the immediate effects of treatments centered around WBV frequency on the balance and walking abilities of 12 children suffering from CP [23]. According to their findings, the immediate effect of WBV demonstrates that the 18 Hz was the most effective in developing walking and balance abilities in children with CP than the 12 or 26 Hz. In the present study, a similar frequency was used. The treatment frequency for all groups was 18 Hz, with an amplitude of 12 mm. The vibration frequency was increased by 1 Hz per two seconds until reaching 18 Hz. Each session consisted of three minutes of vibration therapy followed by two minutes of rest, repeated four times during the 20-minute treatment. Using this protocol, WBVT was found to be beneficial in improving balance and cervical joint position sense in both weight-bearing and non-weight-bearing position for the upper and lower extremities in children with CP.

In our study, the mean PBS score of Group Aa was increased from 25.50 to 28.70 after the intervention of four weeks. In Group Ab, mean score increased from 26.70 to 34.50, and in Group Ac, mean score was 26.20 and after the intervention, it increased to 39.60. In Group Ba, Group Bb, and Group Bc, before the intervention, the mean score was 25.10, 25.50 and 25.00 which was increased to 27.80 31.30 and 35.20, respectively, after the intervention of four weeks. These findings are similar to research by Kim et al. that included 20 mobile individuals suffering from spastic CP [24]. WBVT was performed on an 80 degree inclined table with a vibration frequency of 20 Hz, five times per week for six weeks. Following WBVT training, the participant's PBS increased from 46.00±7.85 to 49.20±6.76, indicating statistical significance (p<0.05). The study found that WBVT is an effective training strategy for regaining balance in spastic CP patients.

Previously, it was proposed that applying the WBVT multiple arrangements, including weight-bearing and non-weight-bearing stances involving the lower as well as upper limbs, might promote brain plasticity via the cross-training effect. It was believed that engaging all limbs in weight-bearing postures would provide more substantial results than focusing primarily on the upper or lower extremities [25]. The findings of our study were according to this hypothesis. WBVT was found to be efficient in improving balance and cervical joint position sense in both weight-bearing and non-weight-bearing position for the upper and lower extremities in children with cerebral palsy. Notably, WBVT for both upper and lower extremities in weight-bearing position consistently showed the most significant improvements in improving both balance and cervical joint position sense, indicating the efficacy of this treatment approach in CP children.

Our study has been limited by several factors. It had a relatively small sample size and it solely focused on hemiplegic children with spastic CP of age 5-15 years, so the findings may not be generalizable to other types of CP or age groups. Moreover, the follow-up period in our study was four weeks which might not be sufficient to capture the long-term effects of WBVT. Future studies with long follow-up assessments and large sample sizes incorporating other types of CP are recommended. It will help to strengthen the evidence base for the effectiveness of WBVT in CP management.

## Conclusions

The results of the present study indicated that WBVT, whether applied to the upper or lower extremities in either weight-bearing or non-weight-bearing positions, leads to substantial improvements in balance and cervical joint position sense. The inter-group comparison showed a statistically significant disparity in all groups, Group Ac receiving WBVT for both upper and lower extremities simultaneously in weight-bearing positions consistently, showed the most significant improvements in both balance and cervical joint position sense, indicating the efficacy of this treatment approach in children with CP.
